# Quantification of computational geometric congruence in surface-based registration for spinal intra-operative three-dimensional navigation

**DOI:** 10.1371/journal.pone.0207137

**Published:** 2019-08-26

**Authors:** Daipayan Guha, Raphael Jakubovic, Michael K. Leung, Howard J. Ginsberg, Michael G. Fehlings, Todd G. Mainprize, Albert Yee, Victor X. D. Yang

**Affiliations:** 1 Division of Neurosurgery, Department of Surgery, University of Toronto, Toronto, Ontario, Canada; 2 Institute of Medical Science, University of Toronto, Toronto, Ontario, Canada; 3 Biophotonics and Bioengineering Laboratory, Sunnybrook Health Sciences Centre, Toronto, Ontario, Canada; 4 Department of Biomedical Physics, Ryerson University, Toronto, Ontario, Canada; 5 Institute of Biomaterials and Biomedical Engineering, University of Toronto, Toronto, Ontario, Canada; 6 Division of Orthopedic Surgery, Department of Surgery, University of Toronto, Toronto, Ontario, Canada; 7 Department of Electrical and Computer Engineering, Ryerson University, Toronto, Ontario, Canada; Mayo Clinic Minnesota, UNITED STATES

## Abstract

**Background Context:**

Computer-assisted navigation (CAN) may guide spinal instrumentation, and requires alignment of patient anatomy to imaging. Iterative closest-point (ICP) algorithms register anatomical and imaging surface datasets, which may fail in the presence of geometric symmetry (congruence), leading to failed registration or inaccurate navigation. Here we computationally quantify geometric congruence in posterior spinal exposures, and identify predictors of potential navigation inaccuracy.

**Methods:**

Midline posterior exposures were performed from C1-S1 in four human cadavers. An optically-based CAN generated surface maps of the posterior elements at each level. Maps were reconstructed to include bilateral hemilamina, or unilateral hemilamina with/without the base of the spinous process. Maps were fitted to symmetrical geometries (cylindrical/spherical/planar) using computational modelling, and the degree of model fit quantified based on the ratio of model inliers to total points.

Geometric congruence was subsequently assessed clinically in 11 patients undergoing midline exposures in the cervical/thoracic/lumbar spine for posterior instrumented fusion.

**Results:**

In cadaveric testing, increased cylindrical/spherical/planar symmetry was seen in the high-cervical and subaxial cervical spine relative to the thoracolumbar spine (p<0.001). Extension of unilateral exposures to include the ipsilateral base of the spinous process decreased symmetry independent of spinal level (p<0.001).

In clinical testing, increased cylindrical/spherical/planar symmetry was seen in the subaxial cervical relative to the thoracolumbar spine (p<0.001), and in the thoracic relative to the lumbar spine (p<0.001). Symmetry in unilateral exposures was decreased by 20% with inclusion of the ipsilateral base of the spinous process.

**Conclusions:**

Geometric congruence is most evident at C1 and the subaxial cervical spine, warranting greater vigilance in navigation accuracy verification. At all levels, inclusion of the base of the spinous process in unilateral registration decreases the likelihood of geometric symmetry and navigation error. This work is important to allow the extension of line-of-sight based registration techniques to minimally-invasive unilateral approaches.

## Introduction

Spinal instrumentation traditionally has been placed freehand based on anatomic landmarks, which may be highly variable, or with fluoroscopic guidance resulting in significant radiation exposure to operating room personnel.[1–3] Computer-assisted navigation (CAN) may guide spinal instrumentation placement, significantly improving accuracy and minimizing acute and long-term malposition related complications.[4–6] Image guidance in CAN may be based on pre-operative imaging, typically CT, or intra-operatively-acquired 3D fluoroscopy or CT; in all cases, navigation requires registration of the image and patient spaces. Our laboratory has developed a novel technique for image-to-patient registration, based on optical topographic imaging (OTI), which rapidly acquires a surface map of exposed spinal anatomy under direct vision and automatically registers to pre-operative CT in real-time, minimizing workflow disturbance.[7–10] Registration of three-dimensional point sets in contemporary surface-based navigation techniques, including OTI, is typically performed using variants of the Iterative closest-point (ICP) algorithm, in which two meshes are aligned using an initial rigid-body pose estimation, followed by iterative refinement of the translational and rotational transformations to minimize a distance error metric between the two meshes.[11,12] ICP algorithms may be prone to instability when too many point pairs arise from unconstrained symmetrical, or congruent, geometries, including cylinders, spheres, and planes.[13–15] While multiple variations of ICP have attempted to address the stability of the final alignment between meshes, non-convergence from geometric congruence remains a potential source of registration error in image-guided surgery, leading to failed registration or, worse, successful registration with inaccurate navigation. ICP convergence is particularly critical in surface-based registration techniques, such as OTI, especially when applied to minimally-invasive (MIS) exposures with fewer available points to increase the variance in input geometry and specify the initial alignment pose.

Here, we therefore quantify geometric congruence, or symmetry, in posterior spinal exposures using computational modelling, and identify predictors of potential navigation inaccuracy from this error mechanism. This understanding is essential to allow the safe and efficient translation of any surface-based navigation technique requiring line-of-sight to exposed anatomy, to minimally-invasive spinal exposures.

## Methods

### Specimen/Patient selection

Surface geometry of posterior spinal exposures was assessed initially in four human cadavers, as part of pre-clinical validation of our OTI navigation system. All cadavers underwent pre-operative helical CT imaging at 0.5mm slice thickness. Institutional ethics board approval was obtained (Mount Sinai Hospital REB# 16-0051-E). Written informed consent for the use of all cadavers in research was provided by the subjects themselves, to the University of Toronto Faculty of Medicine. Cadavers used in our study were accessed only through the University of Toronto Faculty of Medicine.

Surface geometry of posterior spinal anatomy was subsequently assessed *in-vivo* in 11 patients, undergoing midline exposures in the cervical/thoracic/lumbar spine for OTI-guided posterior instrumented fusion as part of an ongoing trial of OTI navigation at Sunnybrook Health Sciences Centre (Sunnybrook Health Sciences Centre REB# 309–2014 and 086–2015). All patients underwent pre-operative helical CT imaging, reformatted at 0.625mm slice thickness.

### OTI registration

**Fig 1:**
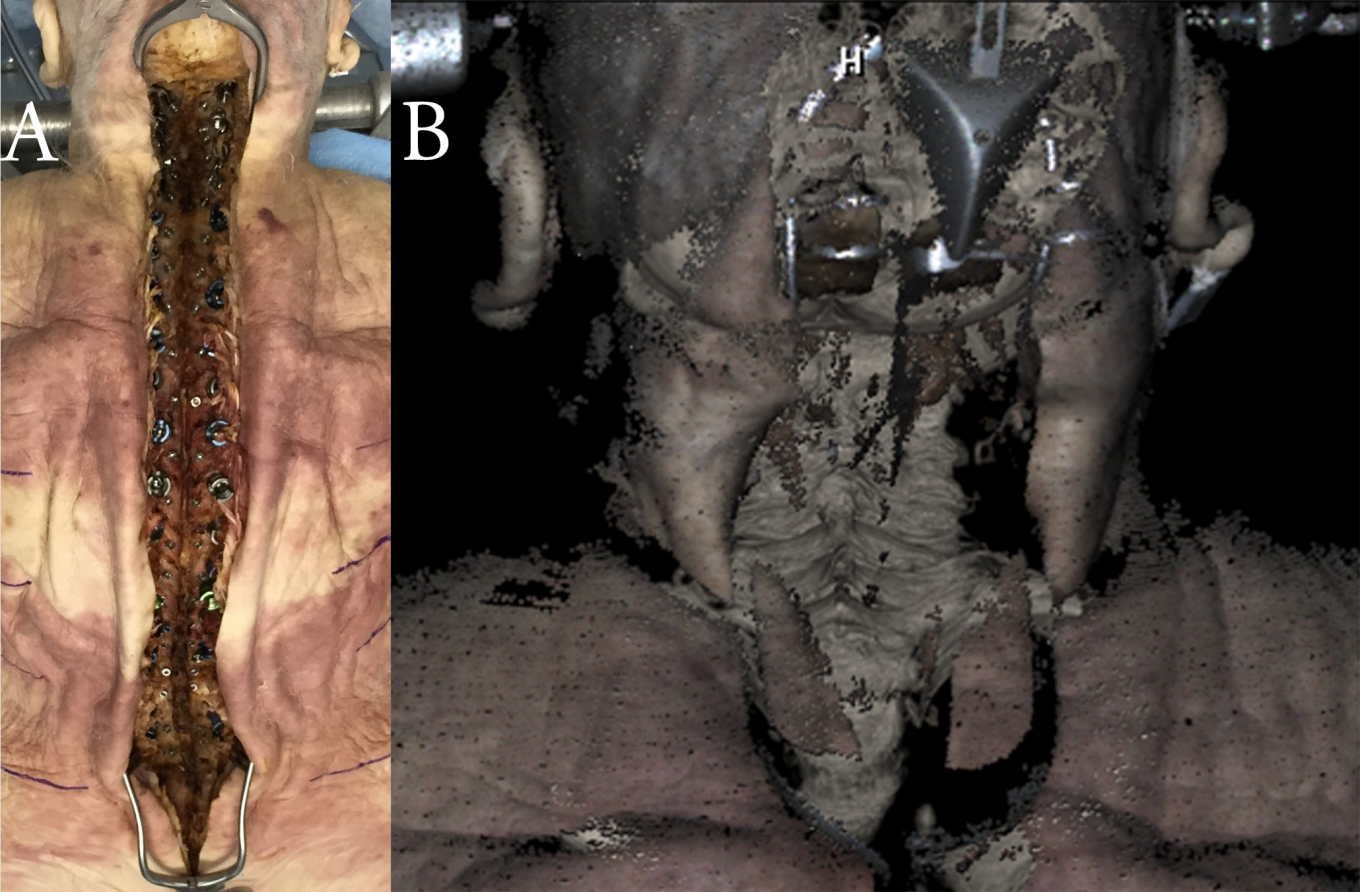
(A) Representative standard midline posterior spinal exposure in cadaveric testing. (B) OTI 3D surface map of a midline posterior cervical spine exposure.

Cadavers were positioned prone on a standard operating table. Midline posterior exposures were performed bilaterally from C1-S1 in four human cadavers, extending to the lateral edge of the lateral masses in the cervical spine, and to the transverse processes in the thoracolumbar spine. 3D surface maps of the posterior elements were generated using OTI at each level (Fig 1). Technical details of OTI registration are described separately.[10] Briefly, structured light is projected onto the exposed anatomy and its deformation recorded by stereoscopic cameras to generate a 3D surface point cloud, followed by automatic registration via an iterative closest-point (ICP) algorithm to the pre-operative CT. Individual vertebrae are automatically segmented following structured-light acquisition to allow level-to-level registration, eliminating confounding from any changes in position from pre-operative CT (supine) to the operative position. This segmentation algorithm also eliminates confounding from pre-existing instrumentation at the surgical site.

In clinical testing, all patients were positioned prone on a Wilson frame. Patients undergoing cervical instrumentation were also placed in a Mayfield head clamp. Standard midline posterior exposures sufficient for open instrumentation placement were performed, with OTI surface acquisition and registration similar to cadaveric testing.

### Computational modelling of geometric congruence

**Fig 2:**
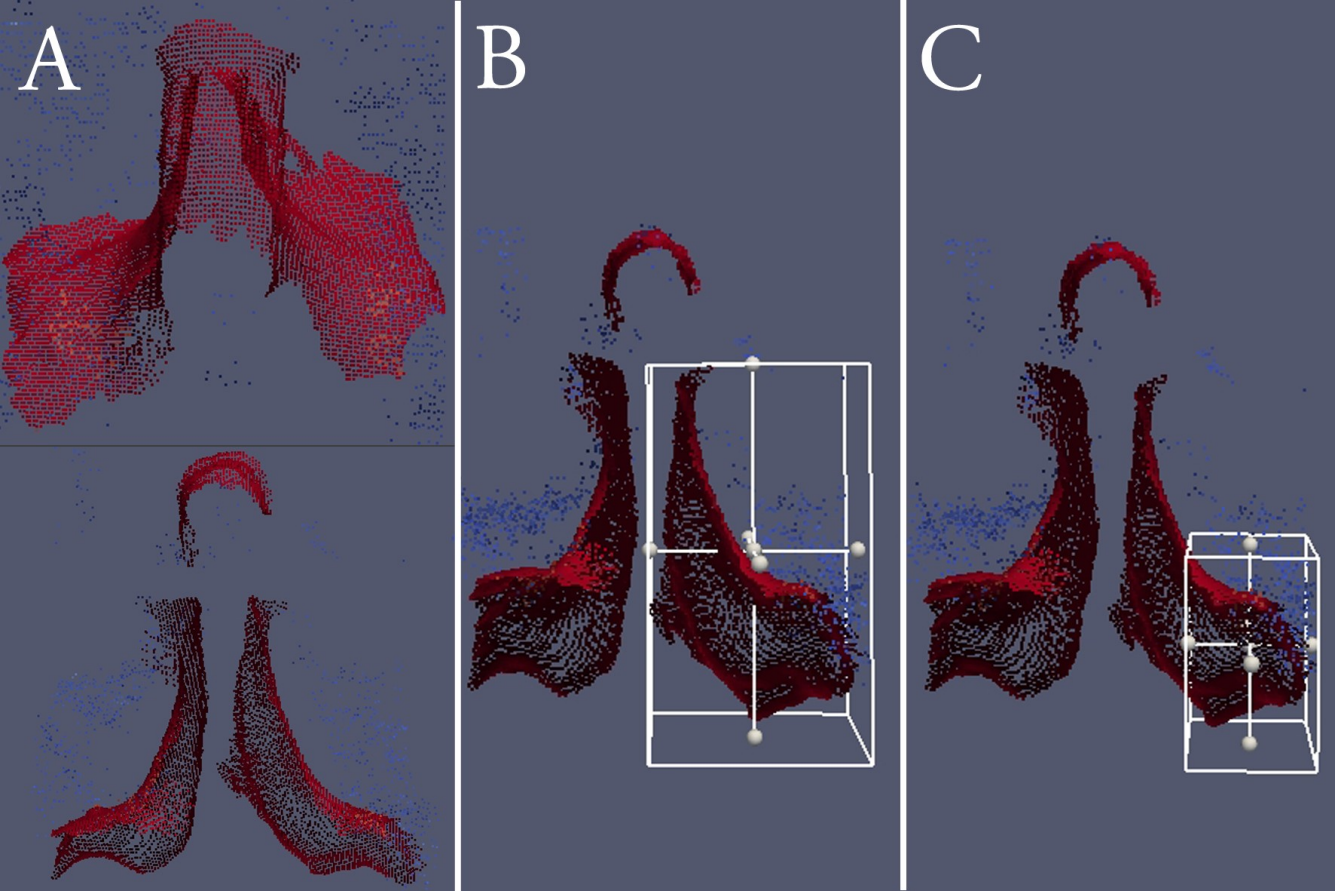
Reconstructed surface map point clouds, capturing the bilateral hemilaminae including spinous process (Group A) viewed from above (top) and axially (bottom), unilateral hemilamina including base of the spinous process (Group B), and unilateral hemilamina excluding the spinous process (Group C).

3D surface maps generated from each vertebral level were thresholded to isolate the vertebra. The point clouds comprising each surface map were subsequently reconstructed to capture the bilateral hemilaminae including spinous process (Group A), each unilateral hemilamina including the ipsilateral base of the spinous process (Group B), and each unilateral hemilamina excluding the spinous process (Group C)(Fig 2). All thresholding and reconstruction was performed in an open-source data visualization package (ParaView 5.2.0. Kitware, Inc.; Clifton Park, NY, USA).

**Fig 3:**
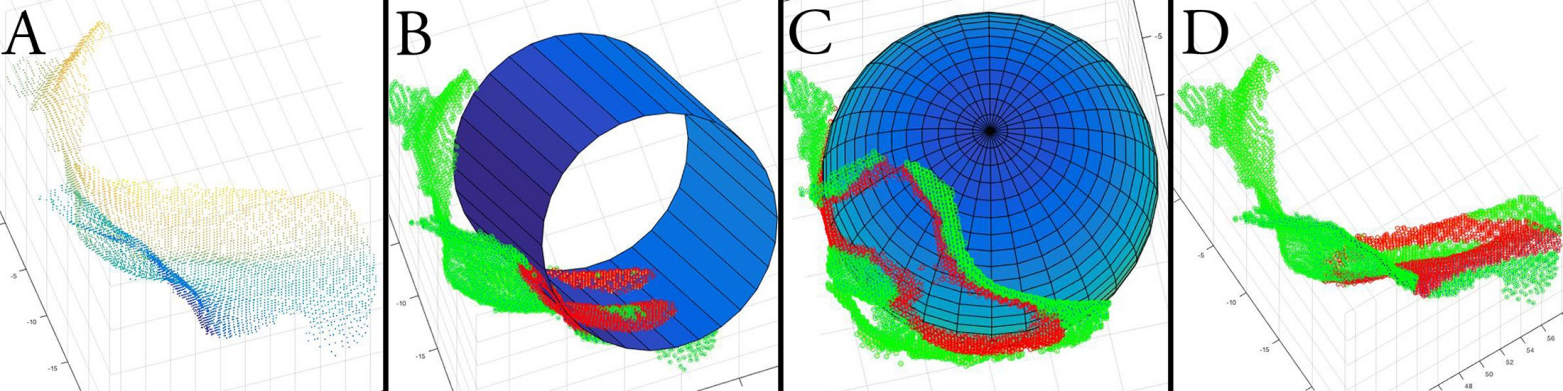
(A) Point cloud of an L2 unilateral hemilamina including base of spinous process (Group B). Fitting of a cylinder (B), sphere (C), and plane (D) to the L2 point cloud. Red dots represent points included in the fitted model (inliers); green dots represent points excluded from the fitted model (outliers).

Reconstructed point clouds from each group at each registered level were subsequently fitted to symmetrical geometries (cylindrical, spherical, planar) in a computing package (MATLAB R2016b. The MathWorks, Inc.; Natick, MA, USA) using a random sample consensus (RANSAC) algorithm, iterated 100 times (Fig 3).

The degree of fit of each point cloud to a geometrically-symmetric shape was quantified using two metrics, the mean-adjusted coefficient of variation in the root-mean-square error (CoV-RMSE) of the point cloud to the fitted shape, as well as the proportion of total points fitted to the symmetric shape (inliers-to-points ratio, ITPR). Increased fit to a symmetric geometry, and therefore increased likelihood of navigation error, is denoted by decreased CoV-RMSE and increased ITPR. As the CoV-RMSE is highly dependent on the user-specified maximum inlier error for the RANSAC algorithm, sensitivity analyses were performed for each geometry with the maximum inlier error set to 0.1mm, 0.5mm, 1.0mm and 2.0mm, and the maximum inlier error resulting in the lowest CoV-RMSE chosen for subsequent ITPR analyses; a maximum inlier error of 0.5mm was selected for all analyses in this study.

### Statistical analysis

Predictors of increased fit to symmetric geometries were explored using univariate and multiple linear regression modelling. For univariate analyses, RMSE and ITPR were compared between spinal levels and between point cloud reconstruction groups using one-way analysis of variance (ANOVA), with Tukey’s Honest-Significant-Difference test for post-hoc comparisons. Differences in CoV-RMSE were compared using Levene’s test of homogeneity of variances. Hierarchical mixed-effects general linear modelling was employed for multivariate analyses to adjust for second-order differences between cadavers/patients. Significance levels for all tests were set at α < 0.05.

All statistical analyses were performed in SPSS Statistics (version 21; IBM. Chicago, IL, USA).

## Results

For the four cadavers used in pre-clinical testing, mean age at death was 91.4 years (range 83–96). *In-vivo* clinical testing was performed in eleven patients, with mean age 58.3 years (range 42–71).

### Geometric congruence by spinal region

**Fig 4:**
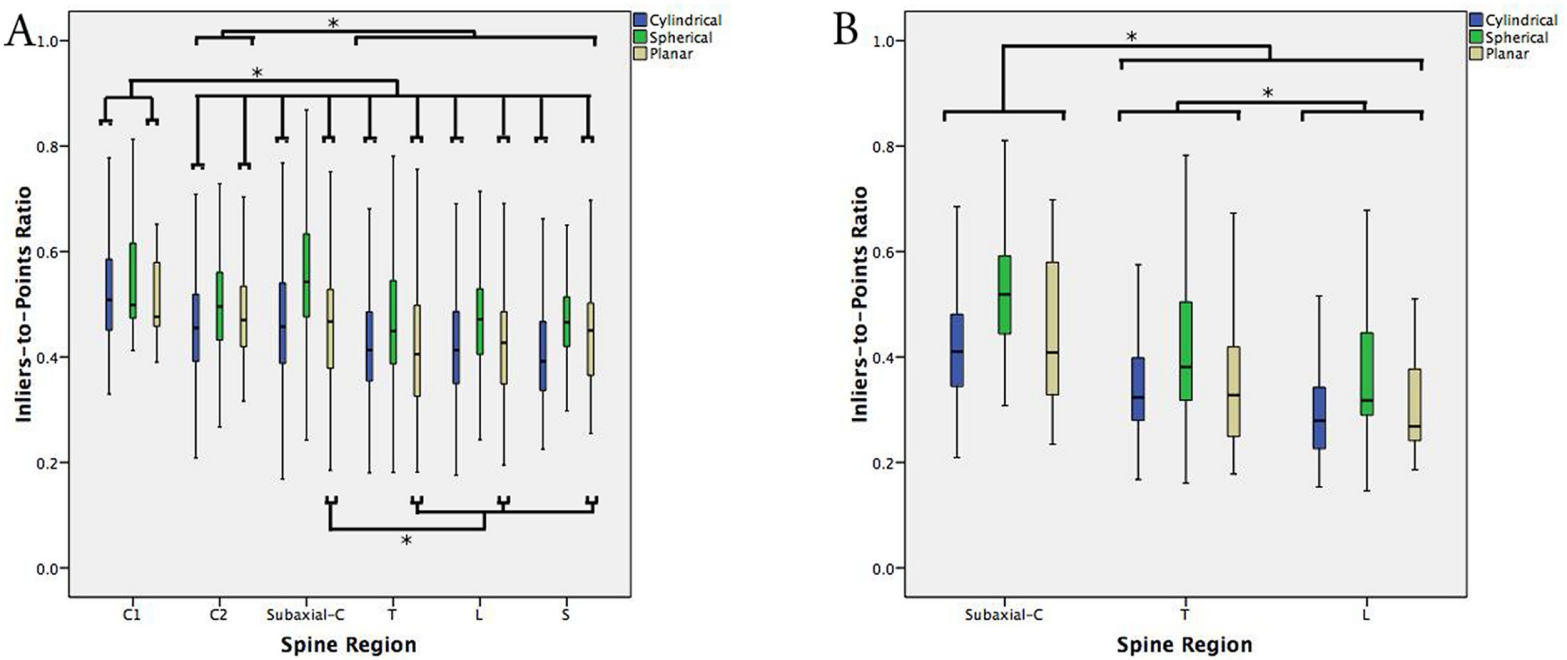
(A) Standard boxplot of the ITPR stratified by spine region (C1, C2, subaxial cervical, T—thoracic, L—lumbar, S—sacrum), for each of cylindrical, spherical and planar geometries, in cadaveric testing. (B) Standard boxplot of the ITPR stratified by spine region, in clinical testing. Error bars represent 1.5xIQR. * denotes significance at p<0.05.

In cadaveric testing, for unilateral registrations (Group C), C1 was found to have greater cylindrical and planar symmetry than C2, the subaxial cervical spine, as well as the thoracic, lumbar and sacral spines, based on ITPR (p<0.001)(Fig 4). C2 demonstrated increased symmetry in all configurations relative to the thoracolumbar and sacral spines, while the subaxial cervical spine showed greater planar symmetry than the thoracolumbar and sacral spines (p<0.001). ITPR stratified by individual spinal levels are shown in Table 1, comparing differences between Groups B and A, and Groups C and B, to demonstrate at which levels a decision to switch from a unilateral to a bilateral exposure, or from a unilateral approach excluding the spinous process to one including it, would be most beneficial for reducing geometric congruence.

**Table 1:** Differences in inliers-to-points ratios (ITPR) between Groups B and A (‘Group B-A’) and Groups C and B (‘Group C-B’) for each symmetrical configuration, stratified by spinal level, in cadaveric testing. All values reported as (mean ± SD).

Level	Cylinder		Sphere		Plane	
	*Group B-A*	*Group C-B*	*Group B-A*	*Group C-B*	*Group B-A*	*Group C-B*
C1	0.146 ± 0.050	0.052 ± 0.009	0.053 ± 0.001	0.052 ± 0.024	0.208 ± 0.087	0.042 ± 0.005
C2	0.192 ± 0.091	0.074 ± 0.015	0.184 ± 0.075	0.060 ± 0.001	0.233 ± 0.111	0.051 ± 0.011
C3	0.160 ± 0.075	0.041 ± 0.021	0.171 ± 0.062	0.047 ± 0.011	0.168 ± 0.090	0.063 ± 0.035
C4	0.095 ± 0.011	0.103 ± 0.032	0.110 ± 0.019	0.107 ± 0.008	0.068 ± 0.004	0.133 ± 0.044
C5	0.202 ± 0.085	0.095 ± 0.021	0.234 ± 0.082	0.078 ± 0.004	0.195 ± 0.090	0.129 ± 0.035
C6	0.212 ± 0.096	0.067 ± 0.016	0.237 ± 0.098	0.050 ± 0.004	0.180 ± 0.070	0.124 ± 0.051
C7	0.215 ± 0.100	0.073 ± 0.022	0.239 ± 0.097	0.051 ± 0.007	0.173 ± 0.087	0.163 ± 0.059
T1	0.208 ± 0.105	0.070 ± 0.015	0.240 ± 0.107	0.068 ± 0.005	0.181 ± 0.095	0.153 ± 0.041
T2	0.200 ± 0.092	0.091 ± 0.020	0.207 ± 0.080	0.110 ± 0.018	0.176 ± 0.077	0.176 ± 0.068
T3	0.160 ± 0.078	0.082 ± 0.029	0.129 ± 0.051	0.108 ± 0.032	0.110 ± 0.046	0.162 ± 0.081
T4	0.159 ± 0.071	0.091 ± 0.033	0.123 ± 0.042	0.122 ± 0.035	0.127 ± 0.055	0.158 ± 0.074
T5	0.167 ± 0.072	0.068 ± 0.026	0.132 ± 0.040	0.095 ± 0.024	0.166 ± 0.063	0.104 ± 0.037
T6	0.171 ± 0.079	0.050 ± 0.026	0.154 ± 0.066	0.055 ± 0.006	0.179 ± 0.082	0.079 ± 0.028
T7	0.144 ± 0.064	0.073 ± 0.030	0.087 ± 0.024	0.113 ± 0.036	0.127 ± 0.048	0.112 ± 0.051
T8	0.159 ± 0.066	0.058 ± 0.026	0.074 ± 0.030	0.123 ± 0.041	0.109 ± 0.041	0.129 ± 0.058
T9	0.147 ± 0.064	0.054 ± 0.033	0.072 ± 0.026	0.111 ± 0.055	0.096 ± 0.040	0.122 ± 0.070
T10	0.173 ± 0.077	0.042 ± 0.019	0.106 ± 0.032	0.056 ± 0.019	0.089 ± 0.023	0.111 ± 0.060
T11	0.189 ± 0.094	0.075 ± 0.019	0.112 ± 0.051	0.142 ± 0.024	0.059 ± 0.026	0.156 ± 0.069
T12	0.184 ± 0.094	0.088 ± 0.025	0.186 ± 0.091	0.109 ± 0.011	0.121 ± 0.065	0.157 ± 0.070
L1	0.189 ± 0.099	0.081 ± 0.028	0.225 ± 0.103	0.097 ± 0.021	0.167 ± 0.084	0.135 ± 0.077
L2	0.192 ± 0.100	0.068 ± 0.029	0.223 ± 0.102	0.081 ± 0.042	0.185 ± 0.088	0.102 ± 0.079
L3	0.203 ± 0.100	0.071 ± 0.019	0.214 ± 0.092	0.074 ± 0.024	0.188 ± 0.080	0.094 ± 0.048
L4	0.224 ± 0.110	0.067 ± 0.022	0.239 ± 0.110	0.064 ± 0.016	0.187 ± 0.086	0.117 ± 0.056
L5	0.213 ± 0.108	0.069 ± 0.014	0.252 ± 0.111	0.082 ± 0.004	0.169 ± 0.092	0.151 ± 0.044
S1	0.096 ± 0.044	0.107 ± 0.042	0.067 ± 0.023	0.130 ± 0.043	0.089 ± 0.050	0.142 ± 0.043

In unilateral *in-vivo* registrations (Group C), the subaxial cervical spine again demonstrated greater symmetry, in all configurations, relative to the thoracic and lumbar spine (p<0.001)(Fig 4). Uniquely, the posterior elements of thoracic vertebrae also showed greater symmetry in all configurations relative to the lumbar spine (p<0.001).

### Geometric congruence by laterality

**Fig 5:**
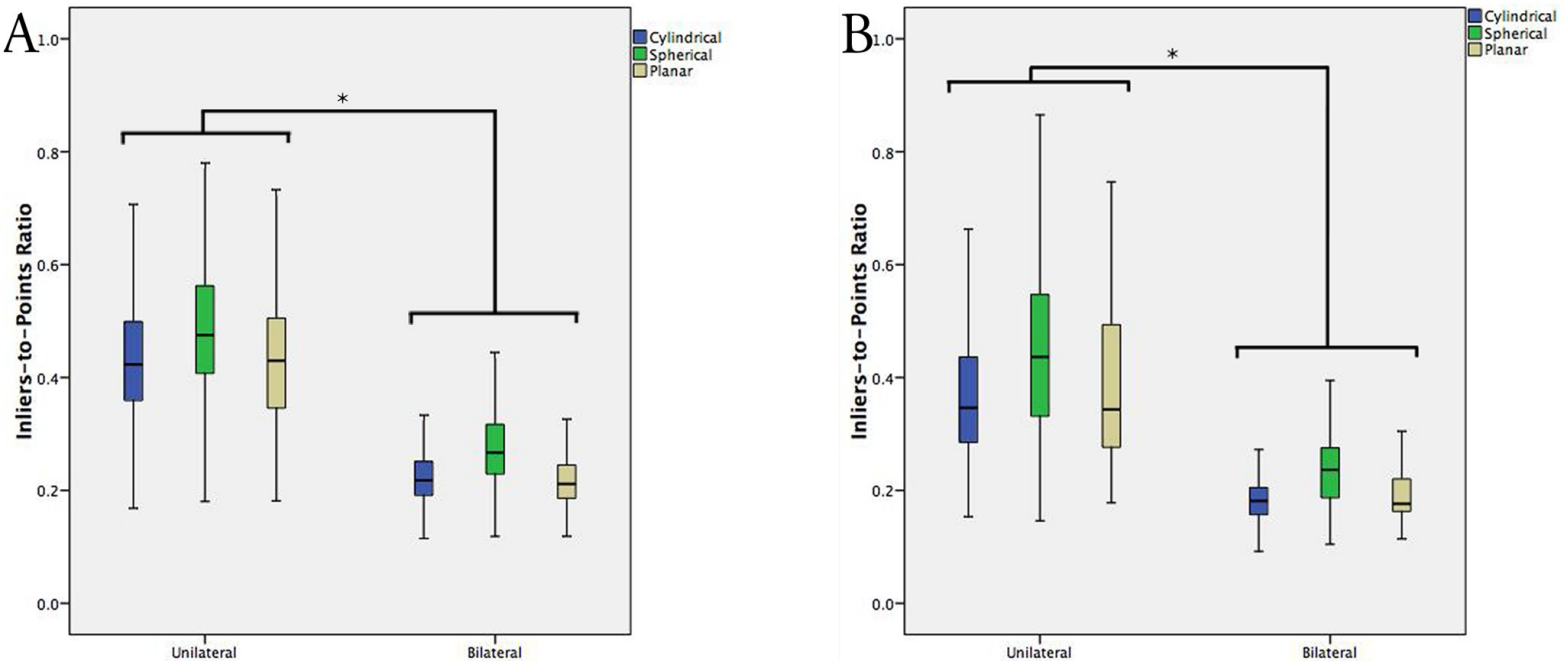
(A) Standard boxplot of the ITPR stratified by unilateral vs. bilateral registrations, for each of cylindrical, spherical and planar geometries, in cadaveric testing. (B) Standard boxplot of the ITPR stratified by unilateral vs. bilateral registrations, in clinical testing. Error bars represent 1.5xIQR. * denotes significance at p<0.05.

**Fig 6:**
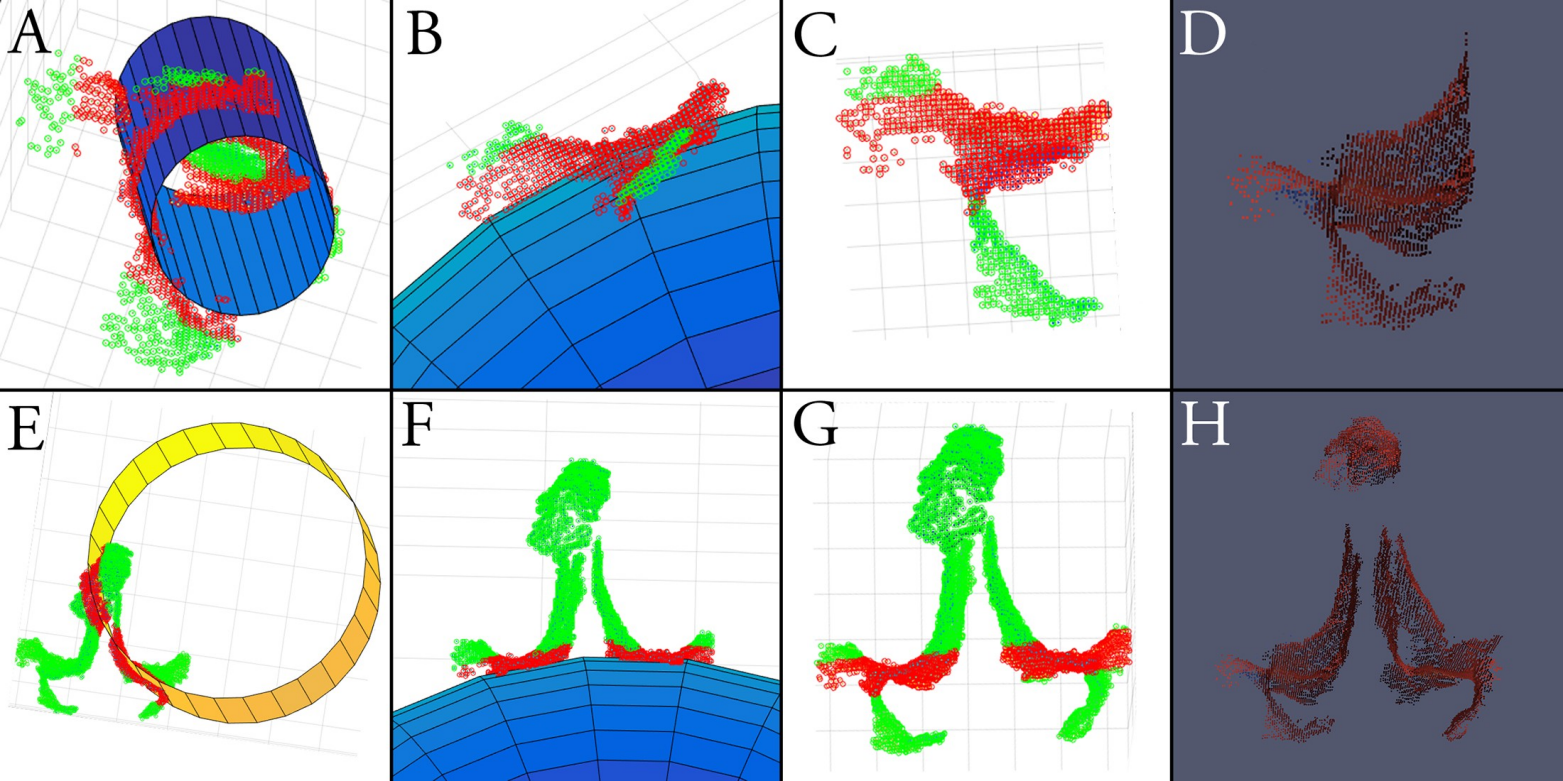
(Top row) Fitting of a cylinder (A), sphere (B), and plane (C) to a unilateral L2 registration (D). Inliers are denoted by red dots, outliers by green dots. (Bottom row) Significant reduction in symmetry, i.e. ratio of inliers (red dots) to outliers (green dots), by extension to a bilateral L2 registration (H), in each of cylindrical (E), spherical (F), and planar (G) geometries.

In cadaveric testing, extension of the registered anatomy from a unilateral exposure (Groups B+C) to a bilateral acquisition (Group A) resulted in significant reduction in symmetry in all geometric configurations (Figs 5 and 6). ITPR for cylindrical configurations, i.e. cylindrical symmetry, was decreased by 47.9% (0.436 ± 0.107 vs. 0.227 ± 0.055, p<0.001)(mean ± SD), spherical symmetry by 42.0% (0.493 ± 0.121 vs. 0.286 ± 0.082, p<0.001), and planar symmetry by 48.6% (0.438 ± 0.119 vs. 0.225 ± 0.063, p<0.001), for unilateral vs. bilateral registrations (Groups B+C vs. Group A).

In clinical testing, absolute ITPRs were decreased for all geometric configurations relative to cadaveric data (p<0.001), but with similar reductions in symmetry by extending unilateral acquisitions (Groups B+C) to a bilateral registration (Group A)(Fig 5). Cylindrical symmetry was reduced by 50.0% (0.366 ± 0.111 vs. 0.183 ± 0.037, p<0.001), spherical symmetry by 47.7% (0.451 ± 0.136 vs. 0.236 ± 0.069, p<0.001), and planar symmetry by 50.8% (0.390 ± 0.144 vs. 0.192 ± 0.048, p<0.001), in Group A vs. Groups B+C.

### Geometric congruence by inclusion of the spinous process

**Fig 7:**
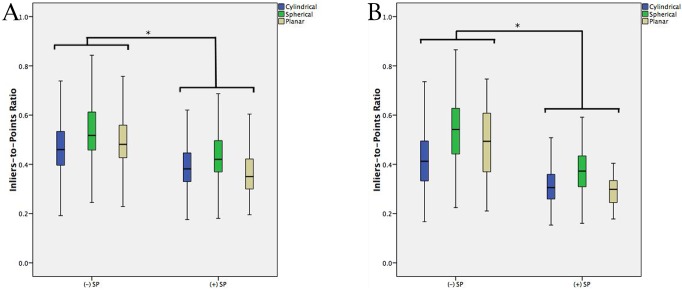
(A) Standard boxplot of the ITPR stratified by inclusion of the spinous process (SP) base, for unilateral registrations, in cadaveric testing. (B) Standard boxplot of the ITPR stratified by inclusion of the spinous process base, in clinical testing. Error bars represent 1.5xIQR. * denotes significance at p<0.05.

**Fig 8:**
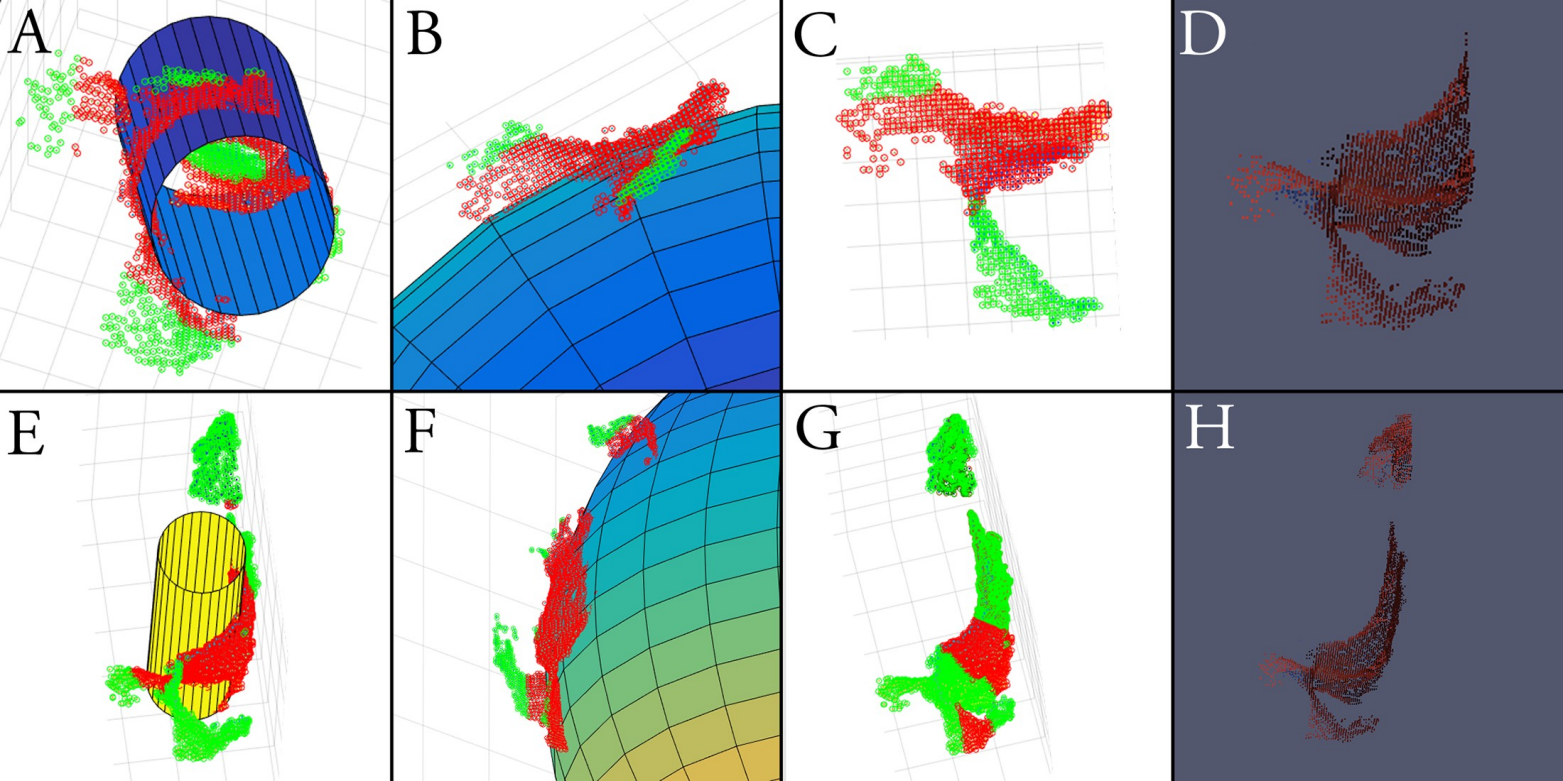
(Top row) Fitting of a cylinder (A), sphere (B), and plane (C) to a unilateral L2 registration excluding the base of the ipsilateral spinous process (D). Inliers are denoted by red dots, outliers by green dots. (Bottom row) Significant reduction in symmetry, i.e. ratio of inliers (red dots) to outliers (green dots), by extension of the unilateral registration to include the base of the ipsilateral spinous process (H), in each of cylindrical (E), spherical (F), and planar (G) geometries.

In cadaveric testing, extension of the registered anatomy from a unilateral exposure (Group C) to include the ipsilateral base of the spinous process (Group B) reduced symmetry significantly in all configurations (Figs 7 and 8). Cylindrical symmetry was reduced by 16.5% (0.472 ± 0.107 vs. 0.394 ± 0.089, p<0.001)(mean ± SD), spherical symmetry by 18.4% (0.539 ± 0.119 vs. 0.440 ± 0.099, p<0.001), and planar symmetry by 26.1% (0.498 ± 0.111 vs. 0.368 ± 0.087, p<0.001), for unilateral registrations including the ipsilateral spinous process base vs. without (Group B vs. Group C).

For *in-vivo* registrations, absolute ITPRs were decreased for all configurations relative to cadaveric testing (p<0.001). Inclusion of the ipsilateral spinous process base in the registration reduced cylindrical symmetry by 24.6% (0.418 ± 0.116 vs. 0.315 ± 0.078, p<0.001), spherical symmetry by 28.8% (0.527 ± 0.136 vs. 0.375 ± 0.082, p<0.001), and planar symmetry by 40.2% (0.488 ± 0.138 vs. 0.292 ± 0.054, p<0.001), relative to registrations excluding the spinous process (Group B vs. Group C).

## Discussion

While CAN has been shown to improve instrumentation accuracy across all spinal levels, widespread adoption has been limited by high capital costs and workflow disruptions.[8,16–21] OTI for spinal navigation significantly streamlines registration workflow by employing rapid optical 3D scanning to generate a high-density surface point cloud.[7] However, OTI requires direct vision of bony anatomy for registration, limiting its applicability to some current paradigms of minimally-invasive approaches. Extension of OTI, and other efficient surface-based registration techniques, to minimally-invasive approaches requires an understanding of mechanisms of registration failure. OTI, along with every current navigation technique applying surface-based registration to pre-operative imaging, employs an ICP algorithm to register point sets. Some pitfalls of ICP algorithms are known, including failed registration due to poor initial pose estimation from large rigid-body fiducial localization errors or soft tissue deformation, susceptibility to mismatched outliers, and inability to account for differences in scale between point sets, resulting in hundreds of variants of the original ICP algorithm published in the past 20 years.[22–26] A lack of convergence, i.e. failed or inaccurate registration, of ICP algorithms in the presence of geometric congruence has been demonstrated in the context of 3D scanned shapes, with multiple variants attempting to minimize the associated rotational error, albeit with a target translational error of <25mm as a definition of ‘successful registration’, far too large for surgical navigation.[15] Other variants have attempted to use a similar RANSAC-algorithm based approach as used in our study, to detect geometric symmetry in the point sets to be aligned, however requiring a computational time of 10 minutes, again unacceptable for real-time surgical navigation.[27] To date, geometric homogeneity has not been demonstrated in the context of surgical navigation; in spinal surgery, geometric congruence is likely to arise in unilateral or minimal-exposure registrations. While CAN techniques employing intra-operative 3D imaging do eliminate this potential error, it comes with significant capital expense, operating time, and workflow hindrance. To allow the workflow improvements of surface-scanning techniques such as OTI to be fully realized, with their significantly greater point density, it is paramount to characterize their potential limitations and failure mechanisms.

Here, we show first that geometric instability, or congruence, is greatest at C1 and in the subaxial cervical spine, in both cadaveric and *in-vivo* settings. This is certainly intuitive given the relatively smooth and symmetric nature of the C1 posterior arch, disrupted minimally by the posterior tubercle. In the subaxial cervical spine, facets are relatively flat and smooth relative to those in the thoracolumbar spine, again resulting in significant geometric congruence that may lead to potential navigation error when registered through minimal exposures. In the literature on navigated pedicle screw placement, breach rates are consistently greater in the cervical spine than in the thoracolumbar spine, although the relatively smaller diameter of cervical pedicles may certainly contribute to this.[20] In our own clinical validation of OTI navigation, quantitative navigation accuracy has been comparable in the cervical vs. thoracolumbar spine, albeit with statistically insignificantly-greater translational and angular errors in the cervical spine.[7,28]

**Fig 9:**
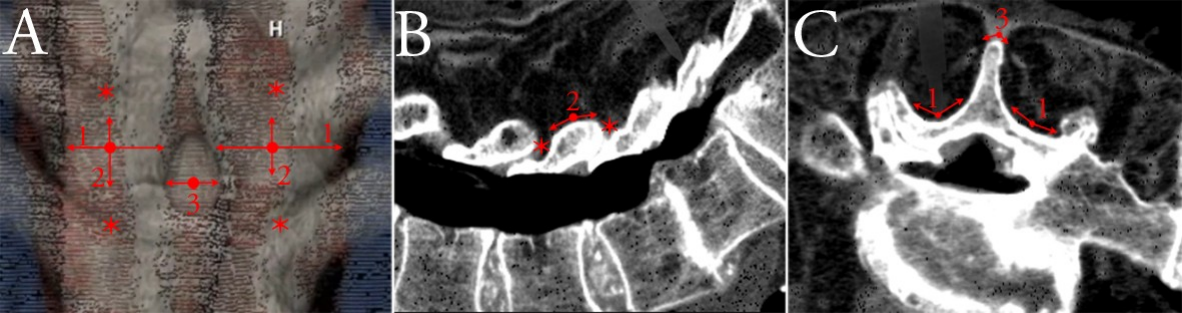
(A) Representative point cloud of an open bilateral posterior lumbar exposure. Manual accuracy verification should be performed with a tracked sharp-tip tool statically at the superior and inferior facet joints (*), and dynamically by sliding axially (1) and sagittally (2) along the hemilaminae as well as along the spinous process tip (3). Verification steps shown on orthogonal sagittal (B) and axial (C) CT reconstructions as seen on typical navigation displays, with static verification points (*) and dynamic sliding maneuvers (1, 2, 3).

For unilateral registrations using surface-based navigation techniques, geometric instability can be improved significantly by extending the registration to a bilateral exposure, intuitive as three-dimensionally-unique geometry in the form of the spinous process and contralateral hemilamina is now included in the surface dataset to be registered by ICP. Similarly intuitively, incorporation of adjacent laminae and therefore multiple vertebral segments in the registration will also improve registration accuracy, though this certainly requires open exposure of these osseous elements for surface-based line-of-sight registration techniques. More practically, however, particularly for minimally-invasive single-level exposures, we show that geometric instability may also be improved in a unilateral registration by simply including the adjacent base of the ipsilateral spinous process in the registered anatomy. While the absolute values of ITPR were greater in our cadaveric vs. clinical testing, due likely to larger and more rounded osteophytes in the significantly older cadaveric population, improvement in symmetry by including the ipsilateral spinous process base was seen in both cadaveric and clinical settings, in fact more so in the *in-vivo* population. Clinically, this has relevance in performing for instance minimally-invasive TLIFs with surface-based navigation guidance, whereby a unilateral exposure is required for the interbody work, and slight medialization of the exposure to include the ipsilateral spinous process base can significantly improve the likelihood of successful navigation registration and implant accuracy. It is important to note, however, that geometric symmetry is minimized but not eliminated with these maneuvers, hence manual verification of navigation accuracy by the surgeon remains paramount to the safe and efficient performance of navigated spinal procedures. We propose a systematic technique of manual registration verification to account for all dimensions in which geometric congruence may lead to navigation error (Fig 9).

Our findings may be extended to surface imaging and navigation for anterior spinal approaches, as well as cranial and non-neurosurgical procedures. Given the lack of spinous processes and other unique geometric landmarks in anterior spinal approaches, navigation registration in this context will likely also be affected by geometric congruence. Inclusion of geometrically-unique degenerative para-discal osteophytes or lateral uncovertebral joints in open anterior approaches will likely minimize registration error in these applications. With further miniaturization of optical topographic imaging techniques to allow registration through endoscopic approaches to the anterior spine, one may speculate that appropriate direction of the endoscope during registration, to include osteophytes or uncovertebral joints, followed by redirection to the pathology of interest, would be a reasonable workflow. In cranial navigation, while initial surface-based registrations prior to patient draping are likely to be accurate due to the unique geometry offered by variant facial features, intra-operative updating of registrations on the external skull requires caution particularly over the convexity, where significant cylindrical and spherical symmetry may be expected.

While our study was conducted using an OTI navigation system, our findings may be generalized to those of any surface-based navigation techniques. Our study is limited by its simulation of unilateral exposures, as our surface scanning was performed on fully-open bilateral exposures, and reconstructed to simulate unilateral registration post-hoc. Future studies of *in-vivo* MIS surgery using tubular retractors are warranted, to assess registration quality in true unilateral exposures.

## Conclusions

Geometric congruence may lead to failed or inaccurate registration with surface-based surgical navigation techniques. Congruence is most evident at C1 and the subaxial cervical spine, warranting greater vigilance in navigation accuracy verification in these regions. At all spinal levels, medial extension of a unilateral exposure to include the base of the ipsilateral spinous process, or to a central bilateral exposure, decreases the likelihood of geometric symmetry and therefore improves the likelihood of successful and accurate navigation in minimally-invasive approaches.

## Supporting information

S1 Dataset (Cadaver) Raw data for inliers-to-points ratios for registration at each spinal level in cadaveric testing. (CSV)

S2 Dataset (Clinical) Raw data for inliers-to-points ratios for registration at each spinal level in clinical testing. (CSV)

## References

[1] Robertson PA, Novotny JE, Grobler LJ, Agbai JU. Reliability of axial landmarks for pedicle screw placement in the lower lumbar spine. Spine (Phila Pa 1976). 1998;23: 60–6. 9460154 10.1097/00007632-199801010-00013

[2] Villard J, Ryang Y-M, Demetriades AK, Reinke A, Behr M, Preuss A, et al Radiation exposure to the surgeon and the patient during posterior lumbar spinal instrumentation: a prospective randomized comparison of navigated versus non-navigated freehand techniques. Spine (Phila Pa 1976). 2014;39 10.1097/BRS.0000000000000351 24732833

[3] Nelson EM, Monazzam SM, Kim KD, Seibert JA, Klineberg EO. Intraoperative fluoroscopy, portable X-ray, and CT: patient and operating room personnel radiation exposure in spinal surgery. Spine J. 2014/06/10. 2014;14: 2985–2991. 10.1016/j.spinee.2014.06.003 24912118

[4] Xiao R, Miller JA, Sabharwal NC, Lubelski D, Alentado VJ, Healy AT, et al Clinical outcomes following spinal fusion using an intraoperative computed tomographic 3D imaging system. J Neurosurg Spine. American Association of Neurological Surgeons; 2017; 1–10. 10.3171/2016.10.SPINE16373 28291408

[5] Luther N, Iorgulescu JB, Geannette C, Gebhard H, Saleh T, Tsiouris AJ, et al Comparison of navigated versus non-navigated pedicle screw placement in 260 patients and 1434 screws: screw accuracy, screw size, and the complexity of surgery. J Spinal Disord Tech. 2015;28: E298–303. 10.1097/BSD.0b013e31828af33e 23511642

[6] Fichtner J, Hofmann N, Rienmüller A, Buchmann N, Gempt J, Kirschke JS, et al Revision Rate of Misplaced Pedicle Screws of the Thoracolumbar Spine—Comparison of 3D Fluoroscopy Navigated with Freehand Placement—A Systematic Analysis and Review of the Literature. World Neurosurg. 2017; 28951183 10.1016/j.wneu.2017.09.091

[7] Jakubovic R, Guha D, Lu M, Gupta S, Cadotte DW, Heyn C, et al A.709: Design and development of a novel, fast, extensive intraoperative registration technique of optical machine vision to pre-operative imaging for cranial and spinal neurosurgical procedures: clinical feasibility and comparison with existing neuronavi. J Neurosurg. 2016;124: A1146–209.27482997

[8] Hartl R, Lam KS, Wang J, Korge A, Kandziora F, Audige L. Worldwide survey on the use of navigation in spine surgery. World Neurosurg. 2012/04/04. 2013;79: 162–172. 10.1016/j.wneu.2012.03.011 22469525

[9] Choo AD, Regev G, Garfin SR, Kim CW. Surgeons’ Perceptions of Spinal Navigation: Analysis of Key Factors Affecting the Lack of Adoption of Spinal Navigation Technology. SAS J. Elsevier; 2008;2: 189–194. 10.1016/SASJ-2008-0007-RR PMC4365663 25802621

[10] Jakubovic R, Guha D, Gupta S, Lu M, Jivraj J, Standish BA, et al High Speed, High Density Intraoperative 3D Optical Topographical Imaging with Efficient Registration to MRI and CT for Craniospinal Surgical Navigation. Sci Rep. 2018;8: 14894 10.1038/s41598-018-32424-z 30291261 PMC6173775

[11] Besi PJ, Mckay ND. A Method for Registration of 3-D Shapes. SPIE—Sens Fusion IV. 1992;1611: 586–606. 10.1117/12.57955

[12] Chen Y, Medioni G. Object modeling by registration of multiple range images. Proceedings 1991 IEEE International Conference on Robotics and Automation. IEEE Comput. Soc. Press; 1991. pp. 2724–2729. 10.1109/ROBOT.1991.132043

[13] Gelfand N, Ikemoto L, Rusinkiewicz S, Levoy M. Geometrically Stable Sampling for the ICP Algorithm. Proc Fourth Int Conf 3-D Digit Imaging Model—IEEE. 2003;

[14] Pottmann H, Hofer M. Geometry of the Squared Distance Function to Curves and Surfaces. Springer, Berlin, Heidelberg; 2003 pp. 221–242. 10.1007/978-3-662-05105-4_12

[15] Armesto L, Minguez J, Montesano L. A generalization of the metric-based iterative closest point technique for 3D scan matching. Proc—IEEE Int Conf Robot Autom. 2010; 1367–1372. 10.1109/ROBOT.2010.5509371

[16] Bourgeois AC, Faulkner AR, Bradley YC, Pasciak AS, Barlow PB, Gash JR, et al Improved Accuracy of Minimally Invasive Transpedicular Screw Placement in the Lumbar Spine With 3-Dimensional Stereotactic Image Guidance: A Comparative Meta-Analysis. J Spinal Disord Tech. 2015;28: 324–9. 10.1097/BSD.0000000000000152 25089676

[17] Tian NF, Huang QS, Zhou P, Zhou Y, Wu RK, Lou Y, et al Pedicle screw insertion accuracy with different assisted methods: a systematic review and meta-analysis of comparative studies. Eur Spine J. 2010/09/24. 2011;20: 846–859. 10.1007/s00586-010-1577-5 20862593 PMC3099151

[18] Hecht N, Kamphuis M, Czabanka M, Hamm B, König S, Woitzik J, et al Intraoperative Iso-C C-Arm Navigation in Craniospinal Surgery: The First 60 Cases. J Neurosurg Spine. 2010;36: E1. 10.1227/01.neu.0000119755.71141.13 15113467

[19] Barsa P, Frőhlich R, Šercl M, Buchvald P, Suchomel P. The intraoperative portable CT scanner-based spinal navigation: a viable option for instrumentation in the region of cervico-thoracic junction. Eur Spine J. 2016; 10.1007/s00586-016-4476-6 26983423

[20] Mason A, Paulsen R, Babuska JM, Rajpal S, Burneikiene S, Nelson EL, et al The accuracy of pedicle screw placement using intraoperative image guidance systems. J Neurosurg Spine. 2013/12/24. 2014;20: 196–203. 10.3171/2013.11.SPINE13413 24358998

[21] Wagner SC, Morrissey PB, Kaye ID, Sebastian A, Butler JS, Kepler CK. Intraoperative pedicle screw navigation does not significantly affect complication rates after spine surgery. J Clin Neurosci. 2017;47: 198–201. 10.1016/j.jocn.2017.09.024 29050896

[22] Clements LW, Chapman WC, Dawant BM. Robust surface registration using salient anatomical features for image-guided liver surgery: Algorithm and validation. 2008; 2528–2540. 10.1118/1.2911920 18649486 PMC2809726

[23] Maurer CR, Aboutanos GB, Dawant BM, Maciunas RJ, Fitzpatrick JM. Registration of 3-D images using weighted geometrical features. IEEE Trans Med Imaging. 1996;15: 836–849. 10.1109/42.544501 18215963

[24] Xin W, Pu J. An Improved ICP Algorithm for Point Cloud Registration. 2010 Int Conf Comput Inf Sci. 2010; 565–568. 10.1109/ICCIS.2010.144

[25] Pomerleau F, Colas F, Siegwart R, Magnenat S. Comparing ICP variants on real-world data sets: Open-source library and experimental protocol. Auton Robots. 2013;34: 133–148. 10.1007/s10514-013-9327-2

[26] Ying S, Peng J, Du S, Qiao H. A scale stretch method based on ICP for 3D data registration. IEEE Trans Autom Sci Eng. 2009;6: 559–565. 10.1109/TASE.2009.2021337

[27] Berner A, Bokeloh M, Wand M, Schilling A, Seidel H-P. A Graph-Based Approach to Symmetry Detection. Symp Vol Point-Based Graph. 2008; 1–8.

[28] Guha D, Jakubovic R, Gupta S, Fehlings M, Yee A, Yang V. 0127: Optical topographic imaging for intraoperative 3d navigation in the cervical spine. Can J Surg. Canadian Journal of Surgery; 2017;60: S104. 10.1097/BSD.0000000000000795 30839418

